# Astrocytes, HIV and the Glymphatic System: A Disease of Disrupted Waste Management?

**DOI:** 10.3389/fcimb.2020.523379

**Published:** 2020-09-29

**Authors:** Caitlin Tice, Jane McDevitt, Dianne Langford

**Affiliations:** ^1^Department of Neuroscience, Lewis Katz School of Medicine, Philadelphia, PA, United States; ^2^Department of Kinesiology, College of Public Health at Temple University, Philadelphia, PA, United States

**Keywords:** NeuroHIV, glymphatic system, brain, astrocyte, neuroinflammation

## Abstract

The discovery of the glial-lymphatic or glymphatic fluid clearance pathway in the rodent brain led researchers to search for a parallel system in humans and to question the implications of this pathway in neurodegenerative diseases. Magnetic resonance imaging studies revealed that several features of the glymphatic system may be present in humans. In both rodents and humans, this pathway promotes the exchange of interstitial fluid (ISF) and cerebrospinal fluid (CSF) through the arterial perivascular spaces into the brain parenchyma. This process is facilitated in part by aquaporin-4 (AQP4) water channels located primarily on astrocytic end feet that abut cerebral endothelial cells of the blood brain barrier. Decreased expression or mislocalization of AQP4 from astrocytic end feet results in decreased interstitial flow, thereby, promoting accumulation of extracellular waste products like hyperphosphorylated Tau (pTau). Accumulation of pTau is a neuropathological hallmark in Alzheimer's disease (AD) and is accompanied by mislocalization of APQ4 from astrocyte end feet to the cell body. HIV infection shares many neuropathological characteristics with AD. Similar to AD, HIV infection of the CNS contributes to abnormal aging with altered AQP4 localization, accumulation of pTau and chronic neuroinflammation. Up to 30% of people with HIV (PWH) suffer from HIV-associated neurocognitive disorders (HAND), and changes in AQP4 may be clinically important as a contributor to cognitive disturbances. In this review, we provide an overview and discussion of the potential contributions of NeuroHIV to glymphatic system functions by focusing on astrocytes and AQP4. Although HAND encompasses a wide range of neurocognitive impairments and levels of neuroinflammation vary among and within PWH, the potential contribution of disruption in AQP4 may be clinically important in some cases. In this review we discuss implications for possible AQP4 disruption on NeuroHIV disease trajectory and how HIV may influence AQP4 function.

## Introduction

Maintaining homeostasis within tissues is dependent on the clearance of excess fluid and interstitial solutes. In the periphery, excess fluid and soluble waste products that include abnormal proteins are removed from the interstitial space and transferred to the lymphatic system that deposits waste products into the systemic circulation for processing by the liver, kidneys, and small intestine for excretion (Liao and Padera, 2013). The density of lymphatic vessels within a tissue is proportional to the metabolic rate of that particular tissue. Despite having a high metabolic rate, the brain is not incorporated into the lymphatic system (Jessen et al., 2015). Waste management in the central nervous system (CNS) is vital for maintaining neuronal connectivity, fitness and crosstalk with other cell types including astrocytes. Since many neurocognitive disorders include accumulation of aberrant proteins and other cellular waste products (Benveniste et al., 2017), understanding mechanisms underlying waste clearance in the brain are critical.

A brain-specific pathway for fluid transport in mice was discovered in 2012 and included the para-arterial influx of subarachnoid cerebrospinal fluid (CSF) into the brain interstitium. This pathway allowed for clearance of interstitial fluid (ISF) along large draining veins (Iliff et al., 2012). In 2015, studies in rodents provided evidence of functional lymphatic-like vessels lining the cranial dural sinuses (Aspelund et al., 2015). This finding challenged the previous notion that the CNS had no lymphatic or lymphatic-like system. In this model, CSF and ISF drain into the glial-lymphatic or glymphatic fluid clearance pathway carrying with it any waste products that have accumulated in the brain (Jessen et al., 2015; Eide et al., 2018). These findings in rodents led researchers to search for a similar pathway in humans. An MRI study in patients with various CSF disorders reported that a CSF tracer drained into cervical lymph nodes (Eide et al., 2018), providing evidence for a glymphatic system in humans. Other studies indicated that the glymphatic system is most active during sleep and plays numerous roles in many biological processes. *In vivo* 2-photon imaging studies in mice have shown that glymphatic activity is greatly increased in a sleep state compared to an awake state due to an influx of CSF that results in a 10% increase in the interstitial space (Sweeney et al., 2019). It is speculated that the increase in glymphatic activity during sleep functions to remove neurotoxic waste that builds up during waking periods (Xie et al., 2013). Moreover, glymphatic function is has been reported to be significantly decreased in experimental rodent models of hemorrhagic and ischemic stroke (Gaberel et al., 2014; Goulay et al., 2017; Schain et al., 2017; Golanov et al., 2018; Rasmussen et al., 2018; Zbesko et al., 2018) and traumatic brain injury (Plog et al., 2015; Lundgaard et al., 2017).

Glymphatic function is proposed to be reduced with aging (Kress et al., 2014), and studies report an 80% decrease in glymphatic function in older mice (18 months) compared to younger mice (2–3 months) (Kress et al., 2014). Thus, it stands to reason that aging is a major risk factor for developing a neurodegenerative disease, many of which are characterized by accumulation of aberrant proteins, such as hyperphosphorylated Tau (pTau). In this context, decreased glymphatic clearance may increase the risk of neurodegenerative changes contributing to cognitive decline in the aging population or in age-related CNS diseases (Rainey-Smith et al., 2018). For example, during aging, cerebral arterial walls begin to stiffen and prevent smooth arterial pulses needed for CSF influx and ISF exchange (Badaut et al., 2014; Sweeney et al., 2019). Given the abnormal aging trajectory in people with HIV (PWH), it is possible that HIV may also negatively impact glymphatic clearance. PWH have normal life expectancies due to the success of combination anti-retroviral therapy (cART) (Cassol et al., 2014) thus, supporting a scenario whereby chronic inflammation associated with viral infection promotes a more rapid or abnormal aging processes. In this review, we will provide an overview and discussion of potential contributions of NeuroHIV to glymphatic system dysfunction and implications for NeuroHIV disease trajectory.

## The Glymphatic System

**Figure 1:**
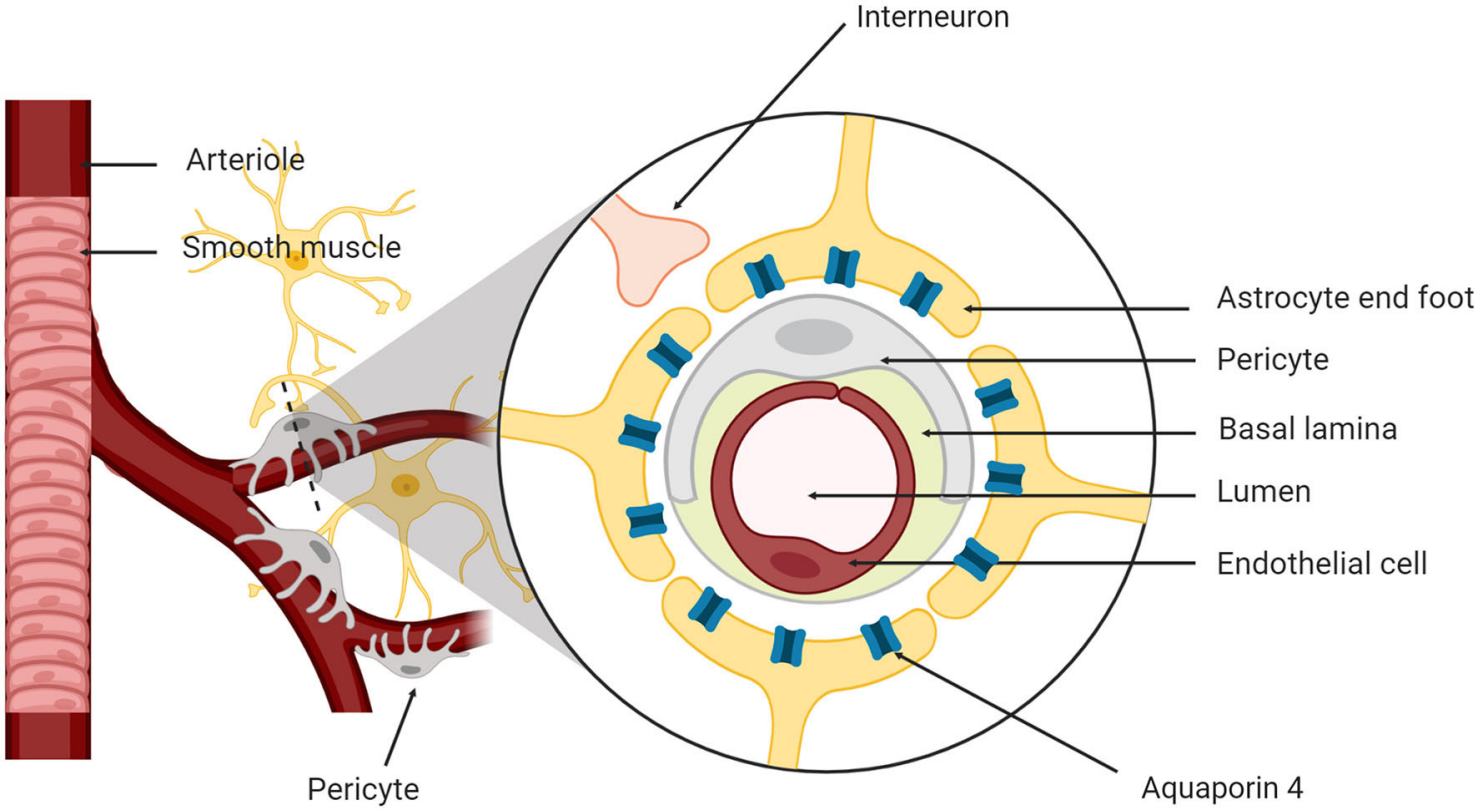
The neurovascular unit. The neurovascular unit is composed of neurons, astrocytes, cerebral endothelial cells, smooth muscle myocytes, pericytes, and matrix components.

Since the discovery of the glymphatic system in both rodents and humans, researchers have focused on understanding the mechanisms of action for CSF and ISF exchange (Mestre et al., 2018). The brain has four fluid compartments including CSF, ISF, intracellular fluid and blood. The blood-brain barrier (BBB) and blood-CSF barrier separate blood from the brain parenchyma and CSF, respectively (Redzic, 2011) to maintain ionic and biochemical composition of the different fluid compartments for proper cellular signaling and brain function (Damkier et al., 2013). The neurovascular unit (NVU) supports brain homeostasis and is composed of neurons, astrocytes, cerebral endothelial cells, myocytes, pericytes and extracellular matrix components (Figure 1; Harder et al., 2002). Cellular components of the NVU detect the changes in CNS cells and signal for necessary responses such as vasodilation and/or vasoconstriction (Koehler et al., 2006).

**Figure 2:**
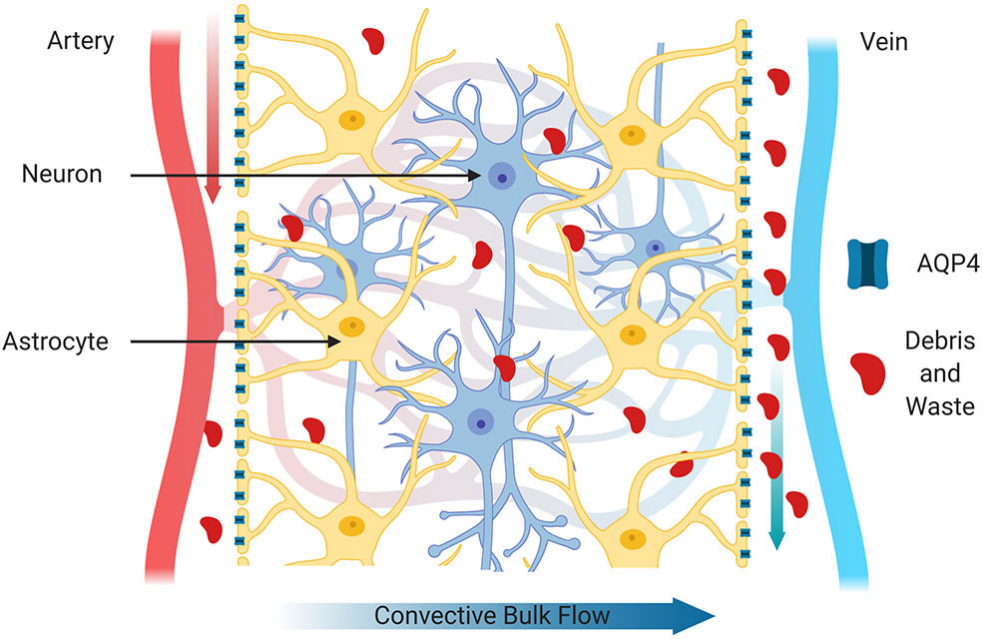
Glymphatic system. The glymphatic system is comprised of astrocytes, aquaporin 4 water channels and cerebral perivascular spaces. Aquaporin 4 water channels, located on astrocytic end feet facilitate movement of water to for the exchange of interstitial fluid and cerebrospinal fluid and to propel extracellular contents such as cellular debris, abnormal proteins and waste products through brain parenchyma for clearance. The glymphatic system also transports glucose, lipids, amino acids, growth factors and neuromodulators through-out the brain. In health, these components can be cleared more efficiently primarily during sleep.

CSF and ISF are constantly exchanged in the brain due to the convective influx of CSF along the periarterial spaces located in the subarachnoid space (Iliff et al., 2012). CSF is then transported to the parenchyma through the NVU by a combination of arterial pulses, respiration, CSF pressure gradients and the loose fibrous matrix of the perivascular space (Figure 2). In addition, water channels called aquaporin-4 (AQP4) located on the perivascular end feet of astrocytes facilitate the transfer of CSF into the parenchyma (Nakada et al., 2017). AQP4 water channels are normally highly concentrated on the perivascular end feet of astrocytes and are critical for the brain's vascular and glymphatic systems. Within the parenchyma, CSF influx promotes CSF/ISF exchange within the peri-venous space that surrounds the deep veins within the brain. ISF then collects in the peri-venous space that is transported out of brain toward the cervical lymphatic system (Figure 2; Iliff et al., 2013).

## Meningeal Lymphatics

The meninges consist of three layers (dura mater, arachnoid, and pia mater) which protect the CNS by lining the brain and spinal cord through forming the various CSF-filled compartments. As with the discovery of the glymphatic system, the CNS and its meningeal components were long believed to lack a traditional lymphatic vascular system since the only lymphatic vessels were identified around the cranial nerves and dural blood vessels (Iliff et al., 2015). Recent studies have described the extensive meningeal lymphatic vessel network that functions in macromolecular clearance and immune cell trafficking in the brain (Aspelund et al., 2015; Louveau et al., 2015). These vessels were found to have immunohistological and structural characteristics of lymphatic vessels, and to express traditional markers of tissue lymphatic endothelial cells, such as Prox1, CD31, Lyve-1, Podoplanin, VEGFR3, and CCL2 (Aspelund et al., 2015; Louveau et al., 2015). Interestingly, meningeal lymphatic vessels are evolutionarily conserved in fish, rats, non-human primates and humans (Absinta et al., 2017; Bower et al., 2017; Jung et al., 2017). Compared to peripheral lymphatics, meningeal lymphatics are composed of a less complex network of thin-walled initial lymphatic vessels that converge and exit the cranium along the retroglenoid vein, sigmoid sinus, and meningeal portions of the pterygopalatine artery (Aspelund et al., 2015; Louveau et al., 2015). Functional studies have shown that meningeal lymphatic vessels can carry numerous immune cells under physiological conditions, suggesting a role in normal immune surveillance of the brain (Aspelund et al., 2015).

Aging is a major risk factor for many neurodegenerative diseases, including Alzheimer's disease (AD) which has a detrimental effect on brain CSF/ISF paravascular recirculation that is thought to be caused by the deterioration of meningeal lymphatic vessels that occurs with aging (Da Mesquita et al., 2018a). Studies have shown that meningeal lymphatic dysfunction in young-adult mice results in impaired brain perfusion by CSF and deficits in learning and memory (Da Mesquita et al., 2018b). Aged mice demonstrated significant disruption of meningeal lymphatic function leading to cognitive decline. Interestingly, augmentation of meningeal lymphatic drainage in aged mice facilitated the clearance of CSF/ISF macromolecules from the brain, resulting in improved cognitive function (Da Mesquita et al., 2018b; Stower, 2018). These data demonstrate that impairment of meningeal lymphatics decreases brain perfusion and leads to diminished cognitive ability accompanied by increased amyloid-beta (Aβ) accumulation. These data suggest that disruption in meningeal lymphatics is at least one of the mechanisms that contribute to the buildup of aberrant proteins observed in AD and other neurocognitive disorders (Da Mesquita et al., 2018b; Stower, 2018).

## Glymphatic and Meningeal Lymphatic Systems Connections

Recent discoveries of the glymphatic and meningeal lymphatic systems have changed the previous idea that the CNS was devoid of lymphatic vessels (Aspelund et al., 2015; Plog and Nedergaard, 2018; Rasmussen et al., 2018). Both the glymphatic and meningeal lymphatic systems allow for the clearance of aberrant proteins such as Aβ and pTau. Thus, both systems are critical for healthy aging as the buildup of aberrant proteins can contribute to AD or other age-related neurocognitive disorders (Rasmussen et al., 2018). The connections between the glymphatic and meningeal lymphatic systems have been demonstrated through studies following tracers injected into the CSF of rodents (Aspelund et al., 2015). Both systems use the water channel, AQP4 to facilitate the movement of CSF throughout the CNS (Filippidis et al., 2016; Cao et al., 2018). The continuous flow of CSF that perfuses the brain parenchyma and is drained to cervical lymph nodes through the meningeal lymphatic system. As previously mentioned, CSF and ISF are constantly exchanged in the brain due to the convective influx of CSF along the peri-arterial spaces located in the subarachnoid space (Iliff et al., 2012). The glymphatic system is responsible for the movement of the CSF into the parenchyma. Within the parenchyma, CSF influx forces connective ISF exchange within the peri-venous space that surrounds the deep veins within the brain (Iliff et al., 2013). The meningeal lymphatic system has been shown to be necessary for the efficient clearance of ISF collected in the subarachnoid space that is transported out of brain toward the deep cervical lymph nodes through dural meningeal lymphatics (Louveau et al., 2015). The deep cervical lymph nodes can connect to the inner carotid and empty the waste metabolites into the general circulation (Aspelund et al., 2015; Louveau et al., 2015). This link provides a plausible route to study CNS-immune connection (Bower and Hogan, 2018). Dysfunction of meningeal lymphatic vessels and their connection to the glymphatic system should be addressed by future studies.

## Aquaporins

Aquaporins (AQPs) are hydrophobic transmembrane proteins that facilitate the passive transport of water depending on the osmotic pressure on both sides of membrane. The functional AQP unit is a homo-tetramer and is comprised of six α-helix transmembrane domains with two conserved asparagine–proline–alanine (NPA) motifs, five loops (A-E) and intracellular N- and C-termini (Potokar et al., 2016). AQPs are subdivided into two subfamilies, classical AQPs that are water selective and aquaglyceroporins that act as glycerol channels. However, this characterization was challenged by recent evidence revealing that both subfamilies overlap functionally, for example, some classical AQPs transport water and other small solutes such as glycerol (Papadopoulos and Verkman, 2013). AQPs are found in all species ranging from simple prokaryotes to complex multicellular organisms. Thirteen AQPs have been identified in mammalian cells with eleven of these in humans. The distribution of AQPs varies among different tissue types (Ma et al., 2011), with AQPs 1, 4, and 9 representing the key aquaporins expressed in the CNS.

## Aquaporins in the Nervous System

AQPs are present in the CNS, spinal cord, enteric nervous system and sensory organs. AQP expression has been studied more extensively in the CNS and sensory organs than in other systems (Rosu et al., 2019). From the three main aquaporins in the CNS, AQP4 has the highest expression levels in the brain and is expressed by astrocytes (Chen et al., 2008). AQP1 and AQP4 are true aquaporins and transport only water, while AQP9 is an aquaglyceroporin that transports both water and glycerol (Papadopoulos and Verkman, 2013). AQPs in the CNS have diverse functions including bidirectional water exchange between the brain and cerebrovascular circulation and CSF for osmoregulation of in the brain. AQPs are also involved in neural cell signal transduction (Badaut et al., 2014; Filippidis et al., 2016).

AQP1 is located on astrocytic cell membrane where it is co-expressed with AQP4. AQP1 is also expressed on the apical surface of the epithelium of the choroid plexus aiding in CSF production and flux (Rosu et al., 2019). AQP1 is not found in normal brain capillary endothelium, however it is highly expressed by peripheral endothelial cells (Rosu et al., 2019). Importantly, AQP1 expression is upregulated in many neurodegenerative disorders including choroid plexus tumors, spinal cord injury, and AD (Filippidis et al., 2016). A recent study showed that AQP1, but not AQP4 expression levels were increased in astrocytes in brains from AD patients (Misawa et al., 2008). Increased AQP1 levels were observed at early stages of AD, and remained elevated throughout more advanced stages of AD (Pérez et al., 2007).

AQP9 is located in the ependymal cells surrounding the cerebral ventricles, brain stem catecholaminergic neurons, dopaminergic neurons of the substantia nigra and ventral tegmental area and astrocytes (Xu et al., 2017). AQP9 is permeable to water and to small solutes including glycerol, urea and monocarboxylates which may contribute to energy metabolism in the CNS (Potokar et al., 2016). AQP9 and AQP4 have similar distribution patterns in mice and rats, suggesting that these two channels may act together to facilitate the movement of water or small solutes between CSF and the brain parenchyma (Yool, 2007; Lv et al., 2018; Shi et al., 2019).

AQP4 is the most abundant AQP in the brain, spinal cord and optic nerve (Potokar et al., 2016), is widely expressed on the plasma membranes of astrocytes with the highest concentration on perivascular end feet of astrocytes (Rosu et al., 2019). In the brain, AQP4 is primarily localized to the sub-pial astrocyte processes that form the glial-limiting membrane, the basolateral membrane of ependymal cells throughout the brain and spinal cord, and the perivascular astrocyte end feet that constitute a main component of the NVU (Hubbard et al., 2018; Ciappelloni et al., 2019; Dasdelen et al., 2019). AQP4 facilitates the flow of water into and out of the brain due its expression on cells that form barriers between the brain and major fluid compartments (Dasdelen et al., 2019).

## Aquaporin 4 Variants

There are six isoforms of AQP4 in astrocytes and some other organ specific cells designated AQP4a-f. The distribution of these isoforms varies among cell types and their cellular location. All of the AQP4 isoforms are expressed on astrocytes either on the plasma membrane or on cellular organelles. AQP4c is also expressed in the kidney and the sarcolemma of fast-twitch fibers of skeletal muscle (Frigeri et al., 1998; Potokar et al., 2016). AQP4a and AQP4c are most commonly located in the plasma membranes of perivascular end feet of astrocytes, along with AQP4e that is expressed at much lower levels. AQP4c is the predominant isoform located on the plasma membrane compared to AQP4a (Hubbard et al., 2018).

Interestingly, a recent study reported that a single nucleotide polymorphism (SNP) in AQP4, rs72878776, is associated with altered overall sleep quality according to the Pittsburgh Sleep Quality Index (PSQI) sleep parameters such as sleep latency, comfort, and difficulty falling asleep (Rainey-Smith et al., 2018). Individuals that are homozygous for the AQP4 rs72878776-A (0.001% of the population) allele reported worse overall sleep compared to those with a different genotype (Rainey-Smith et al., 2018). This SNP is in the 5-prime untranslated region (5′UTR) of the AQP4 gene and may be of functional relevance through potentially influencing gene transcription, via modification (creation or deletion) of transcription factor binding sites (Rainey-Smith et al., 2018). Individuals with AQP4 variant rs72878776 may be at greater risk for neurodegenerative diseases due to impaired glymphatic function accompanied by poor sleep quality (Rainey-Smith et al., 2018). In addition, individuals homozygous for either AQP4 SNPS rs3763040-A or rs3763043-A have been reported to experience more rapid cognitive decline after AD diagnosis (Burfeind et al., 2017). Other noncoding AQP4 SNPs were associated with altered rates of cognitive decline after AD diagnosis. Interestingly, individuals homozygous for either AQP4 SNPS rs9951307-A and rs3875089-A have been reported to experience slower cognitive decline (Burfeind et al., 2017). These studies indicate the importance of AQP4 in maintaining proper glymphatic clearance in age-related neurodegenerative diseases in both protective and detrimental aspects.

## Aquaporins and Neuroinflammation

Astrocytes express all of the AQPs (1, 4, and 9) found in the CNS. Astrocytes are the primary homeostatic cells of the brain and are the only cell type in the CNS that can undergo rapid volume changes (Thrane et al., 2014). The CNS must tightly regulate water homeostasis as the brain is confined within the skull allowing limited space for expansion. AQP4 is the main water channel in the brain thus, it is critical that AQP4 functions properly (Papadopoulos and Verkman, 2013). This is increasingly important given that injury can decrease expression and induce mislocalization of AQP4 from end feet to cell body, thereby reducing fluid exchange. As extracellular volume changes, ion concentration is also changed to alter neuronal excitability (Dalkara et al., 2011). Disturbances in water homeostasis in the CNS are typically observed in CNS diseases/disorders including HIV infection, AD, PD, meningitis, stroke, brain abscesses, tumors or neurotrauma, all of which have a neuroinflammatory component. It has been established that expression of AQP4 is pro-inflammatory (Chmelova et al., 2019). For example, studies demonstrate increased neuroinflammation in wild type mice administered LPS compared to APQ4 knockout mice (Wu et al., 2019). Astrocytes from wild type mice produced higher levels of the pro-inflammatory cytokines TNFα and IL-6 in response to LPS than AQP4 knockout mice. These data suggest that neuroinflammation is dependent, in part on AQP4 (Zhang et al., 2019). AQP4 is also the target antigen in the neurological autoimmune disease neuromyelitis spectrum disorder, which is an autoimmune astrocyte-specific cytopathy characterized by astrocyte loss, demyelination in the spinal cord, optic nerve and brain (Hinson et al., 2008; Sakalauskaite-Juodeikiene et al., 2018; Ciappelloni et al., 2019; Mader and Brimberg, 2019). Neuromyelitis spectrum disorder patients have chronic neuroinflammation leading to the classic presentations of this disease which include long segments of spinal cord inflammation, severe optic nerve inflammation, and area postrema syndrome (Huda et al., 2019). Area postrema syndrome results from inflammation in the emetic reflex center located in the rhomboid fossa of the 4th ventricle leading to nausea, vomiting, and hiccups in patients (Duvernoy and Risold, 2007). All neuromyelitis spectrum disorder patients are treated with AQP4 antibodies at their first attack with long-term immunosuppression (Mealy et al., 2014).

## AQP4 Dysregulation, Adenosine A2a Receptor and Neurodegeneration

Adenosine A2a receptor (A2aR) is highly expressed by astrocytes and microglia and contributes significantly to neuroinflammatory and neuromodulator processes (Orr et al., 2015; Zhao et al., 2017; Masjedi et al., 2019). Many neurodegenerative diseases including HIV, AD and TBI involve chronic neuroinflammation thereby increasing the risk for regional accumulation of pTau, tau oligomers and neurofibrillary tangles contributing to neurocognitive dysfunction. The substantial neuroinflammatory response may contribute to disruption of the glymphatic system in part, through dysregulation of AQP4 (Iliff et al., 2014; Rasmussen et al., 2018). In fact, in a rodent model of TBI, disruption of perivascular polarization of AQP4 from astrocyte end feet to the soma promoted tau pathology (Xu et al., 2017; Zhao et al., 2017). Widespread reactive astrogliosis was observed 7 days after TBI in multiple brain regions, and impaired AQP4 polarity correlated with regions of pTau accumulation and reactive astrogliosis (Zhao et al., 2017). In this context, a significant increase in A2aR expression was detected 1 day, 3 days, 7 days, and 4 weeks post-TBI (Zhao et al., 2017). This is important because A2aR activates the adenylate cycle to generate cAMP that may contribute to AQP4 mislocalization (Borroto-Escuela et al., 2018). A2aR knockout alleviated the disruption of AQP4 polarity, pTau accumulation and neuronal damage post-injury (Zhao et al., 2017). However, the mechanism(s) of how A2aR/cAMP mediate AQP4 mislocalization is not clear and further studies are warranted in this direction.

A recent study showed increased expression of A2aR in the hippocampus of AD patients compared to aged-matched controls (Orr et al., 2015). Moreover, in an AD-model, mice expressing the human amyloid precursor protein (hAPP), increased expression of A2aR was observed in astrocytes (Orr et al., 2015). However, A2aR receptor ablation enhanced memory in older hAPP mice even when there was a significant abundance of amyloid plaques. In studies of mouse models of TBI, localization of AQP4 to the cell soma and was accompanied by increased accumulation of Tau in this region. However, 1 month post-TBI, pTau accumulated in the brain parenchyma with continued deterioration of AQP4 polarity (Zhao et al., 2017). In these studies, increased levels of A2aR was accompanied by the mislocalization of AQP4 (Zhao et al., 2017) and possibly increased accumulation of aberrant proteins in TBI. These data were supported by further data showing that inactivation of A2aR prevented TBI induced AQP4 mislocalization and pTau accumulation.

Although no literature exists that investigate expression of A2aR in PWH, there are numerous overlapping neuropathological hallmarks between HIV and age-related neurodegenerative diseases that may involve A2aR. For example, progressive accumulation of aberrant proteins such as pTau, Aβ and α-synuclein are accompanied by damage to selective neural circuits, neuroinflammation, gliosis and vascular alterations are common in both HIV and AD (Mackiewicz et al., 2019). In fact, the HIV protein Tat that may be released from infected astrocytes, even during effective anti-retroviral therapy, is believed to be a major factor in promoting pTau and Aβ formation and accumulation (Hategan et al., 2019). Taken together, even though limited, current evidence indicates that changes in AQP4 localization on astrocytes and decreased waste clearance may involve increased A2aR activity. Potential contributions of HIV infection of the CNS to APQ4 mislocalization in astrocytes, astrocyte phenotype and impaired waste clearance and clearly need to be explored. In this context, it is important to understand interactions among HIV, astrocytes and other cells in the brain.

## Astrocytes and HIV

HIV-infected monocytes/macrophages or CD4+ T-cells can cross the BBB and release virions or viral proteins that may negatively impact other cells types in the brain (Valcour et al., 2011). Perivascular macrophages and microglia are the primary cell types in the CNS that are infected by HIV-1 and support productive viral replication (Rojas-Celis et al., 2019). Neurons and oligodendrocytes are not believed to be infected by HIV-1. HIV infection of the CNS occurs early after infection and macrophages and microglia are responsible in large part for productive infection within the CNS. HIV has been shown to infect astrocytes to a lesser degree, and productive infection and release of infectious virions by astrocytes has not been observed consistently (Brack-Werner et al., 1992; Conant et al., 1994; Brack-Werner, 1999; Canki et al., 2001; Olivier et al., 2018). Astrocytes are not infected with HIV through classical gp120 and CD4 binding, but possibly through cell-to cell contact or receptor mediated endocytosis (Chauhan et al., 2014; Do et al., 2014; Luo and He, 2015; Russell et al., 2017), allowing for reverse transcription and incorporation of viral DNA into the host genome (Narasipura et al., 2012). Taken together, most data support that astrocytes can be infected by HIV, release high levels of inflammatory factors and produce several viral proteins including Tat, Nef, and Rev, which promote inflammation and damage to surrounding cells (Ferrell and Giunta, 2014; Hong and Banks, 2015). In fact, recent studies propose that astrocytes infected with HIV in addition to releasing viral proteins, may also release infectious virions that can infect T-cells in the brain that can then be trafficked to the periphery (Lutgen et al., 2020).

## Astrocyte Phenotype and Glymphatics

**Figure 3:**
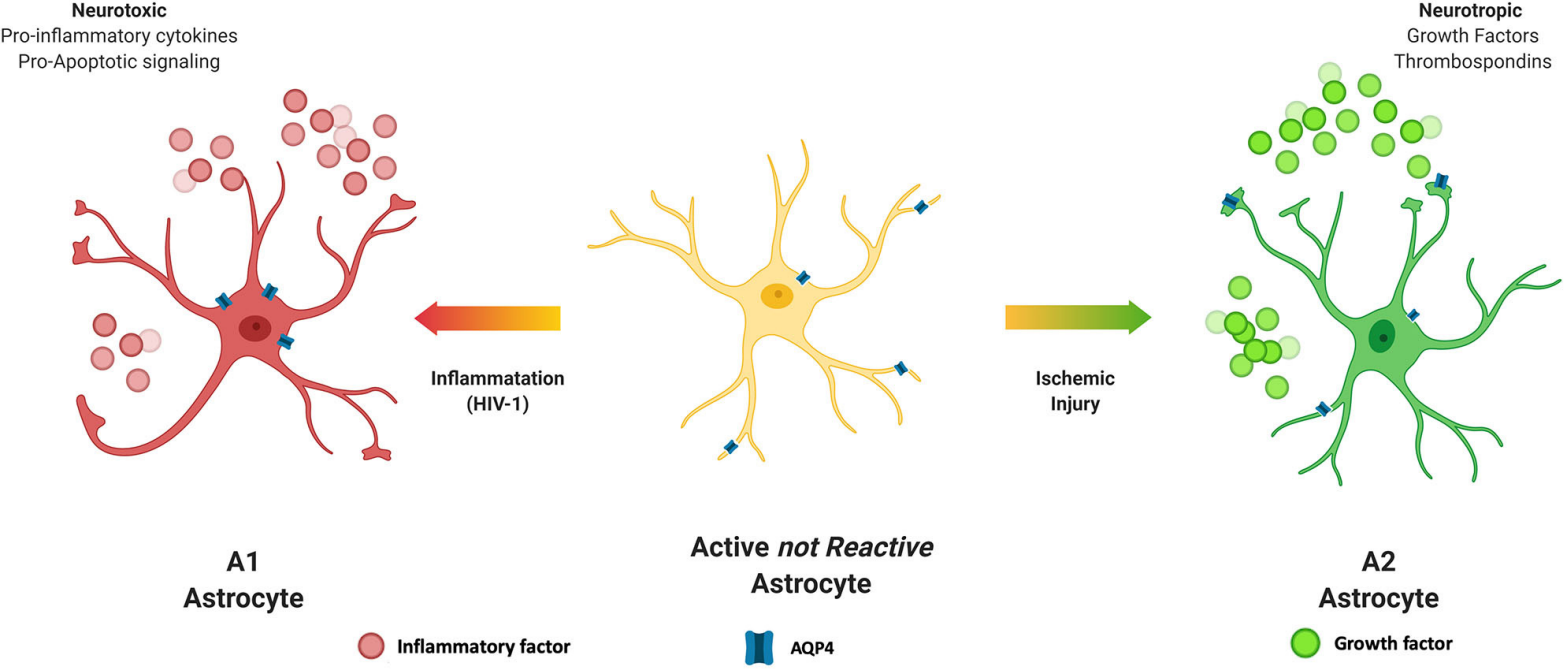
Astrocyte subtypes. Astrocytes can be classified into numerous subtypes based on morphology, active state and anatomical location. Astrocytes are stimulated to become reactive by challenges such as inflammation or ischemia. The A1 subtype is induced by inflammation, is generally considered neurotoxic and produces inflammatory cytokines and pro-apoptotic factors. On the other hand, the A2 subtype is induced by ischemic conditions, such as stroke and releases neurotrophic factors and is considered to be neuroprotective.

Astrocytes make up about 30% of the cells in the CNS (Churchill et al., 2015) and in health, play vital roles in many homeostatic mechanisms to maintain proper CNS functioning and to provide trophic support of neurons (Sun et al., 2019). Thus, it stands to reason that during HIV infection of the CNS, the proper functioning of astrocytes whether infected by the virus as described above, or activated by chronic neuroinflammation would be impacted. Rationale for this prediction is supported by studies from other neurodegenerative diseases with chronic inflammation and reactive glial cell populations. Upon stressful stimuli, astrocytes become activated from a quiescent state, become reactive and undergo structural and functional changes (Ferrer, 2017; Hinkle et al., 2019). In this context, astrocytes can be classified into numerous subtypes based on morphology, activation state and anatomical location. For the purpose of this review, we will consider two reactive subtypes, A1 and A2 that are induced by inflammation or ischemia, respectively (Zamanian et al., 2012; Liddelow et al., 2017; Joshi et al., 2019; Figure 3). The A1 subtype has increased complement 3 (Liddelow et al., 2017; Cohen and Torres, 2019), a neuroinflammatory profile, and releases NFκB-related inflammatory cytokines including TNF-α and IL-1β. Transcriptome analyses of reactive astrocytes show that A1 neuroinflammatory reactive astrocytes upregulate many genes that are destructive to synapses (Shijo et al., 2019). A1 astrocytes are more common in CNS HIV infection and neurodegenerative disease including AD (Cohen and Torres, 2019) and are specifically associated with neurotoxicity. For example, studies demonstrate that amyloid plaques and pTau in the brains of AD patients are surrounded by A1 astrocytes (Li et al., 2011).

The A2 subtype on the other hand, is induced by ischemic event. Ischemia-induced A2 reactive astrocytes upregulate many neurotrophic factors that promote survival and growth of neurons. The A2 subtype is associated with a neurotrophic profile with increased levels of BDNF, FGF2 and VEGF to promote CNS repair and regeneration (Gao et al., 2005; Zador et al., 2009; Hayakawa et al., 2014). A2 astrocytes also produce thrombospondins that aid in synapse repair (Chan et al., 2019).

Since A1 and A2 phenotypes may impact glymphatic function differently, consideration should be given to neurodegenerative events that are accompanied by increased accumulation of aberrant proteins or waste products that are characterized by one phenotype or the other. For example, the A1 phenotype is most commonly observed in HIV, AD, PD, epilepsy, and TBI, all of which include increased accumulation of aberrant proteins or waste products, as well as chronic inflammation (Liddelow and Barres, 2017; Clark et al., 2019; Matias et al., 2019). Interestingly, normal aging as well as age-related neurodegeneration are accompanied by the increased presence of A1 astrocytes (Clarke et al., 2018). Given that AQP4 is reported to be mislocalized in the A1 phenotype, it may be surmised that this could lead to glymphatic dysregulation. On the other hand, the A2 phenotype is generally reported to be protective, however recent studies report that brain and BBB ischemia, and reperfusion can promote changes in Tau that include both increased and decreased phosphorylation (Pluta et al., 2018a,b). Given that astrocytic phenotype has been described as existing in a heterogeneous continuum of mixed populations, more research is needed to determine how glymphatic function in the context of a specific clinical setting is impacted.

As discussed in previous sections of the review, some astrocytes are infected by HIV and can produce and release several viral proteins such as Tat, Nef, and Rev due to integrated virus, thereby inducing an A1 phenotype and production of inflammatory factors that damage surrounding cells (Hinkle et al., 2019; Shijo et al., 2019). Thus, the A1 astrocyte phenotype likely contributes in part to the development HAND (Ances and Clifford, 2008).

## HIV-Associated Neurocognitive Disorder (HAND)

With the development of cART, PWH typically have normal life spans (Bandera et al., 2019). While cART has dramatically increased the life expectancy of PWH, it has also brought attention to an array of neurocognitive issues, collectively known as HAND. HAND has been described as a spectrum disorder that is typically diagnosed by neurocognitive testing to assess attention, information processing, language, executive functioning, sensory-perceptual skills, simple motor skills, complex perceptual-motor skills and memory (Olivier et al., 2018; Robinson-Papp et al., 2019; Saloner et al., 2019). In earlier efforts, the National Institute of Mental Health and National Institute of Neurological Diseases and Stroke charged a working group to assess HAND criteria and develop an updated consensus for the definition (Antinori et al., 2007). According to this criteria in what is commonly called the “Frascati criteria,” the following three conditions are recognized: asymptomatic neurocognitive impairment (ANI), mild neurocognitive disorder (MND), and HIV-associated dementia (HAD) (Antinori et al., 2007). ANI is currently the most prevalent manifestation of HAND in the developing world (Métral et al., 2020) and can be difficult for clinicians to diagnose as the deterioration of cognitive functioning is undetectable by some assessments. Importantly, research indicates that ANI is clinically important because these individuals are at risk for transitioning to the more severe forms of HAND (Ciccarelli, 2019). Participants of the CNS HIV Antiretroviral Therapy Effects Research (CHARTER) study who had ANI at baseline were two to six times more likely to develop symptomatic HAND during several years of follow-up than those who had no impairment at baseline (Olivier et al., 2018; Ciccarelli, 2019). MND is similar to that of ANI also may causes difficulty for diagnosis (Haddow et al., 2013). Like ANI, MND may also be difficult diagnose in PWH when they are one standard deviation below mean in two cognitive domains based from the Frascati Criteria. HAD is the most severe of the form of HAND and according tot he Frascati Criteria is diagnosed in PWH when they are two standard deviations below the mean in at least two cognitive domains (Olivier et al., 2018; Cysique and Brew, 2019; Matchanova et al., 2019). Despite the evolution of defining and diagnosing HAND over the past several decades, debate over screening tools and management plans continue (Winston and Spudich, 2020). In fact, numerous contributing factors to cognitive impairment in PWH must be considered and include, nearly normal life spans with advanced age, age-related co-morbidities, effects of initial infection on the CNS, lifestyle, medications other than cART, cART-induced toxicities, and chronic neuroinflammation (Winston and Spudich, 2020), all of which my impact astrocyte reactivity and waste clearance from the brain.

## Altered Waste Clearance and Abnormal Aging in HAND

**Figure 4:**
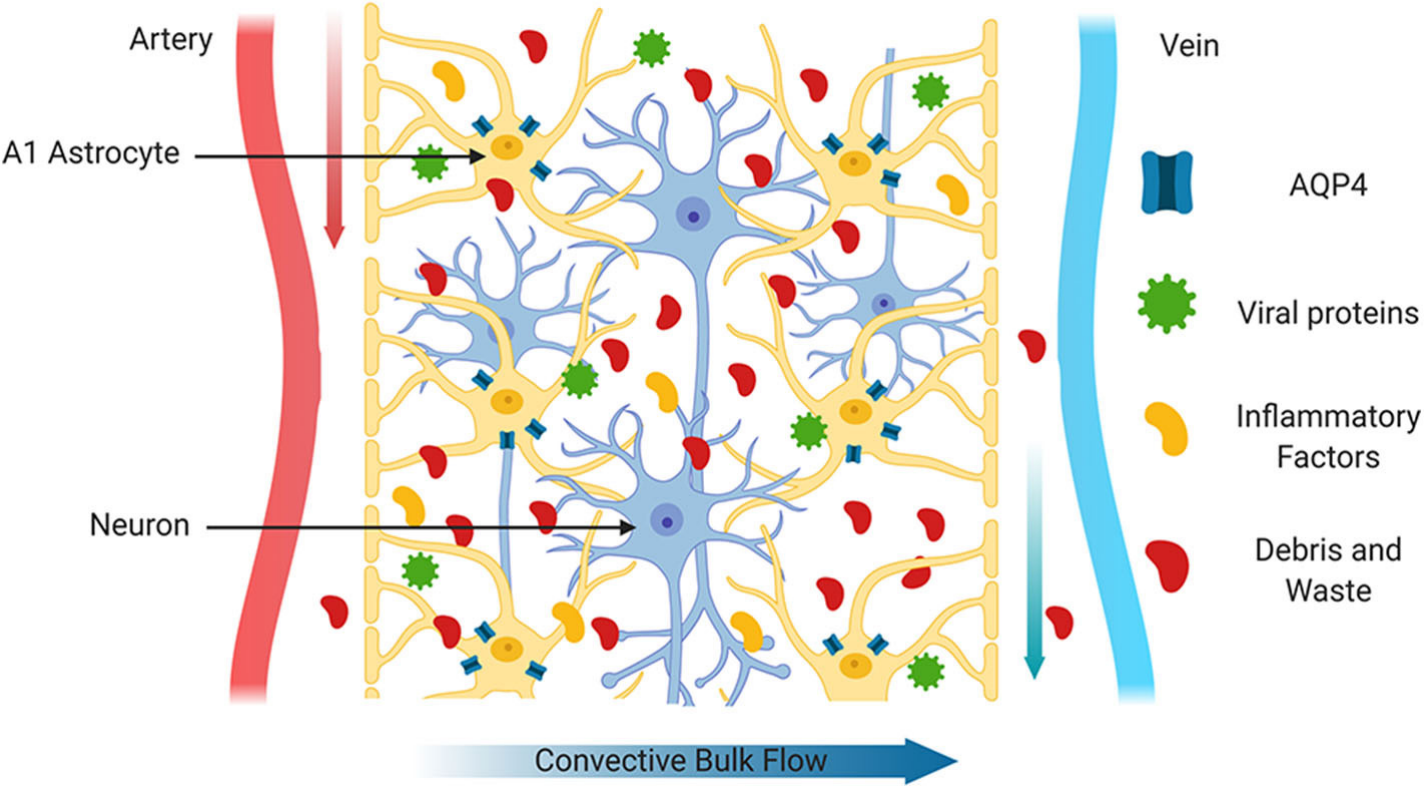
Impaired glymphatic system. After injury or during CNS diseases with a neuroinflammatory component such as HIV infection, the glymphatic system functions less efficiently. This may be due in part to increased inflammatory factors or to the production of viral proteins by HIV infected cells. In this scenario, mislocalization of AQP4 from astrocyte end feet to the cell body and/or reduced expression of AQP4 may lead to accumulation of toxic waste products such as hyperphosphorylated Tau.

The prevalence of PWH over 50 years of age is increasing rapidly each year, and is predicted to increase from 28 to 73% by 2030 (Yang et al., 2019). There is growing evidence that the prevalence of comorbidities and other age-related conditions including geriatric syndromes, functional or neurocognitive problems, polypharmacy or social difficulties are higher in PWH than in their uninfected counterparts (Guaraldi et al., 2019; Winston and Spudich, 2020). Limited information is available regarding the optimal clinical management of older PWH (Chen et al., 2019; Guaraldi et al., 2019; Guo and Buch, 2019). Chronic neuroinflammation has been associated with altered synaptic connectivity and BBB function, and with neuronal injury (Bandera et al., 2019; Guaraldi et al., 2019). In the CNS, age-associated neuroinflammation is also linked with impaired waste clearance (Cassol et al., 2014). In a larger metabolomic profiling study comparing CSF from PWH on cART to CSF from age matched HIV negative individuals, alterations in the HIV CSF metabolome indicated persistent inflammation, glial reactivity, and glutamate neurotoxicity (Cassol et al., 2014). Moreover, abnormal aging in PWH impacts brain waste disposal systems and likely contribute to mechanisms involved in HAND (Cassol et al., 2014). Importantly, these alterations were not directly associated with cART or with ongoing HIV replication in CSF or plasma (Cassol et al., 2014). Although specific mechanisms that could be directly associated with HAND were not identified, it has been speculated that HIV-mediated disruption of AQP4 may contribute to altered glymphatic function (Xing et al., 2017; Figure 4).

## Cervical Lymph Nodes as a Reservoir for HIV

As described above, CSF and ISF exchange occurs in the peri-venous space and ISF is then transported from the brain to the cervical lymph nodes (Figure 2; Iliff et al., 2013). Cervical lymph nodes (CLNs) are a group of lymph nodes in the neck region that are located adjacent to the cervical region of the spinal cord near the sternocleidomastoid muscle (Dave et al., 2018a). The glymphatic and meningeal systems function as a waste removal pathway by connecting the CNS to the CLNs (Benveniste et al., 2017). T cells and antigen-presenting cells (APCs) that are present within the CNS drain via the lymphatic path into the CLNs (Benveniste et al., 2017). CLNs contain follicular dendritic cells (FDCs) which can retain antigens for a prolonged duration allowing them to potentially act as a reservoir for HIV (Sabri et al., 2016; Dave et al., 2018a). FDCs are important for immune function as they allow for germinal center formation, long term immune recovery (Dave et al., 2018a,b) and may contribute significantly to immune system response to the virus, the viral reservoir and escape. Importantly, given the intimate connection between glymphatic drainage and CLNs, the potential role of CLNs in glymphatic impairment and HIV infection should be considered. In addition to well-studied viral reservoirs including macrophages, microglia and astrocytes (Churchill et al., 2015), virion-immune complexes may become trapped within the interconnected processes of FDCs, and may represent a significant reservoir for the virus (Fletcher et al., 2014; Dave et al., 2018a). Studies also show that infectious virus can be recovered from the FDCs PWH who are cART experienced, indicating that FDCs (Ho et al., 2007; Fletcher et al., 2014; Heesters et al., 2015). FDCs have been also been shown to retain infectious virus inside endosomes even in the presence of neutralizing antibodies (Heesters et al., 2015). On the other hand, in several species of non-human primates (NHP) infected with simian immunodeficiency virus (SIV), progression to disease including chronic inflammation, usually does not occur (Rey-Cuillé et al., 1998; Diop et al., 2000; Goldstein et al., 2000; Broussard et al., 2001; Onanga et al., 2002). Additionally, other studies have shown that FDCs do not serve as reservoirs for SIV in NHP (Paiardini and Muller-Trutwin, 2013; Huot et al., 2016, 2018). NHP hosts control viral replication in lymph nodes and resolve the inflammatory response despite high viremia. Although no studies have investigated potential glymphatic impairment in SIV-infected NHP, evidence for a similar system has been reported whereby subarachnoid stroke resulted in impaired CSF flux into periarterial regions (Goulay et al., 2017). Given the striking differences between SIV-infected NHP and HIV infection of humans in viral reservoirs, viral and inflammation control in the context of CLN response, studies aimed at AQP4 and astrocytic involvement in SIV are warranted.

## Aquaporin 4 and NeuroHIV

Few studies have investigated changes in AQP4 and HIV CNS infection. In one study, expression levels of AQP4 in brain homogenates from the mid frontal gyrus of HAD patients and to a lesser extent HIV patients without HAD, were significantly increased (St Hillaire et al., 2005). Immunolabeling of brain tissue indicated patchy AQP4 in perivascular regions as well as in morphologically characterized reactive astrocytes not necessarily associated with brain vasculature.

A single study in the SIV-infected macaque model using three different viruses, SIVmac239, simian-human immunodeficiency virus (SHIV)-RT and SIVsm543-3 assessed changes in levels and patterns of AQP4 in the frontal cortex (Xing et al., 2017). In uninfected control brains, AQP4 was diffusely and evenly expressed in subpial and perivascular areas of the cortex, suggesting expression on astrocytic cell bodies as well as processes. On the other hand, in the brains of infected animals, AQP4 labeling was largely limited to astrocyte-like cells with loss of even distribution. Areas showing loss of AQP4 labeling showed intense GFAP labeling with intracellular dense glial fibrils and increased pro-apoptotic caspase-3 expression (Xing et al., 2017). Thus, these findings support the concept that viral infection does in fact alter patterns and levels of AQP4 in the brain (Xing et al., 2017). Importantly, patterns of AQP4 in SIV brains were reminiscent of those observed in HIV patients' brains (St Hillaire et al., 2005; Xing et al., 2017). Studies by Aoki-Yoshino *et al* report that HIV encephalitis and HAD are not the only neuroinflammatory diseases with altered AQP4 expression and report changes in progressive multifocal leukoencephalopathy and multiple sclerosis (Aoki-Yoshino et al., 2005).

## Summary

As mentioned previously, the brain was initially thought to be devoid of typical lymphatic system, but in the past few years, studies have indicated that there is a system whereby CSF and ISF interchange to clear waste products, solutes and maintain homeostatic conditions within the brain parenchyma. The glymphatic system has been shown to clear aberrant proteins including pTau, Aβ and other macromolecules that tend to increase and accumulate in neurodegenerative diseases. Proper functioning of the glymphatic system is therefore particularly important in age-related CNS diseases including AD and HAND, both of which are characterized by glial cell activation, chronic inflammation and accumulation of toxic waste products. It is also important in recovery from TBI, whereby secondary injury cascades lead to accumulation of pTau, chronic inflammation, reactive gliosis in addition to structural damage. Astrocyte expression of the water channel AQP4 is intimately involved in normal glymphatic clearance and its function is impaired in AD, TBI and HIV infection of the brain. The proper localization of AQP4 in end foot processes of astrocytes is essential to its function (Figure 2), and in neurodegenerative diseases with chronic inflammation, increased ROS and glial cell reactivity, AQP4 becomes localized to the cell body (Figure 4).

Thus far, most studies of glymphatic impairment and AQP4 mislocalization have been conducted in AD and TBI patients or animal models. Given the neuropathological similarities among AD, TBI and HIV infection of the CNS, investigations into the potential impact of viral latency and reactivation in the brain are particularly timely. For example, AQP4 mislocalization on astrocytes and decreased waste clearance may involve increased A2aR-mediated cAMP generation, especially since blocking A2aR activity preserved AQP4 localization to astrocyte end feet accompanied by decreased pTau accumulation.

Among the important considerations in proposing that HIV infection of the brain may be impacted by glymphatic disruption are the changing status and spectrum of HAND within a person with HIV during their lifetime. In this context, astrocyte reactivity, phenotype and neuroinflammatory status can fluctuate. Another critical aspect of potential glymphatic involvement in HIV infection is the role played by CLNs in human infection compared to SIV infected NHP. Given that LN FDCs may serve as a reservoir in human infection with limited immune control, and peri-venous collection of ISF drainage into CLNs that may have lost their normal architecture and be harboring virus, another level of complexity exists within studies aimed at understanding if and how HIV impacts glymphatic and lymphatic functioning.

Approaches aimed at improving AQP4 functioning in the brains of PWH may include modulating activation levels of A2aR and assessing flux of fluid through the brain parenchyma. Studies to investigate molecular changes and neurobiological consequences of regulating A2aR are necessary both *in vitro* and *in vivo*. Positron emission tomography (PET) imaging studies have shown reduced clearance of CSF in AD patients and in patients with normal pressure hydrocephalus (De Leon et al., 2017; Ringstad et al., 2017, 2018). In this context, early detection of changes in clearance using PET imaging may be possible. Consideration must also be given to interactions among the dural lymphatic network, CLN and glymphatic clearance can be linked. Only after increased understanding of disease-specific alterations in these pathways can potential new therapeutic directions be realized.

## Author Contributions

CT wrote manuscript and created figures. JM wrote manuscript. DL wrote manuscript and edited the manuscript. All authors contributed to the article and approved the submitted version.

## Conflict of Interest

The authors declare that the research was conducted in the absence of any commercial or financial relationships that could be construed as a potential conflict of interest.

## References

[B1] Absinta M. Ha S. K. Nair G. Sati P. Luciano N. J. Palisoc M.. (2017). Human and nonhuman primate meninges harbor lymphatic vessels that can be visualized noninvasively by MRI. Elife 6:e29738. 10.7554/eLife.29738 28971799 PMC5626482

[B2] Ances B. M. Clifford D. B. (2008). HIV-associated neurocognitive disorders and the impact of combination antiretroviral therapies. Curr. Neurol. Neurosci. Rep. 8, 455–461. 10.1007/s11910-008-0073-3 18957181 PMC3932327

[B3] Antinori A. Arendt G. Becker J. T. Brew B. J. Byrd D. A. Cherner M.. (2007). Updated research nosology for HIV-associated neurocognitive disorders. Neurology 69, 1789–1799. 10.1212/01.WNL.0000287431.88658.8b 17914061 PMC4472366

[B4] Aoki-Yoshino K. Uchihara T. Duyckaerts C. Nakamura A. Hauw J. J. Wakayama Y. (2005). Enhanced expression of aquaporin 4 in human brain with inflammatory diseases. Acta Neuropathol. 110, 281–288. 10.1007/s00401-005-1052-2 16133546

[B5] Aspelund A. Antila S. Proulx S. T. Karlsen T. V. Karaman S. Detmar M.. (2015). A dural lymphatic vascular system that drains brain interstitial fluid and macromolecules. J. Exp. Med. 212, 991–999. 10.1084/jem.20142290 26077718 PMC4493418

[B6] Badaut J. Fukuda A. M. Jullienne A. Petry K. G. (2014). Aquaporin and brain diseases. Biochim. Biophys. Acta 1840, 1554–1565. 10.1016/j.bbagen.2013.10.032 24513456 PMC3960327

[B7] Bandera A. Taramasso L. Bozzi G. Muscatello A. Gori A. (2019). HIV-associated neurocognitive impairment in the modern ART Era: are we close to discovering reliable biomarkers in the setting of virological suppression? Front. Aging. Neurosci. 11:187. 10.3389/fnagi.2019.00187 31427955 PMC6687760

[B8] Benveniste H. Lee H. Volkow N. D. (2017). The glymphatic pathway: waste removal from the CNS via cerebrospinal fluid transport. Neuroscientist 23, 454–465. 10.1177/1073858417691030 28466758 PMC5547012

[B9] Borroto-Escuela D. O. Hinz S. Navarro G. Franco R. Müller C. E. Fuxe K. (2018). Understanding the role of adenosine A2AR heteroreceptor complexes in neurodegeneration and neuroinflammation. Front. Neurosci. 12:43. 10.3389/fnins.2018.00043 29467608 PMC5808169

[B10] Bower N. I. Hogan B. M. (2018). Brain drains: new insights into brain clearance pathways from lymphatic biology. J. Mol. Med. 96, 383–390. 10.1007/s00109-018-1634-9 29610928

[B11] Bower N. I. Koltowska K. Pichol-Thievend C. Virshup I. Paterson S. Lagendijk A.. (2017). Mural lymphatic endothelial cells regulate meningeal angiogenesis in the zebrafish. Nat. Neurosci. 20, 774–783. 10.1038/nn.4558 28459441

[B12] Brack-Werner R. (1999). Astrocytes: HIV cellular reservoirs and important participants in neuropathogenesis. AIDS 13, 1–22. 10.1097/00002030-199901140-00003 10207540

[B13] Brack-Werner R. Kleinschmidt A. Ludvigsen A. Mellert W. Neumann M. Herrmann R.. (1992). Infection of human brain cells by HIV-1: restricted virus production in chronically infected human glial cell lines. AIDS 6, 273–285. 10.1097/00002030-199203000-00004 1373627

[B14] Broussard S. R. Staprans S. I. White R. Whitehead E. M. Feinberg M. B. Allan J. S. (2001). Simian immunodeficiency virus replicates to high levels in naturally infected African green monkeys without inducing immunologic or neurologic disease. J. Virol. 75, 2262–2275. 10.1128/JVI.75.5.2262-2275.2001 11160730 PMC114810

[B15] Burfeind K. G. Murchison C. F. Westaway S. K. Simon M. J. Erten-Lyons D. Kaye J. A.. (2017). The effects of noncoding aquaporin-4 single-nucleotide polymorphisms on cognition and functional progression of Alzheimer's disease. Alzheimers Dement. 3, 348–359. 10.1016/j.trci.2017.05.001 29067342 PMC5651426

[B16] Canki M. Thai J. N. F. Chao W. Ghorpade A. Potash M. J. Volsky D. J. (2001). Highly productive infection with pseudotyped human immunodeficiency virus type 1 (HIV-1) indicates no intracellular restrictions to HIV-1 replication in primary human astrocytes. J. Virol. 75, 7925–7933. 10.1128/JVI.75.17.7925-7933.2001 11483737 PMC115036

[B17] Cao X. Xu H. Feng W. Su D. Xiao M. (2018). Deletion of aquaporin-4 aggravates brain pathology after blocking of the meningeal lymphatic drainage. Brain Res. Bull. 143, 83–96. 10.1016/j.brainresbull.2018.10.007 30347264

[B18] Cassol E. Misra V. Dutta A. Morgello S. Gabuzda D. (2014). Cerebrospinal fluid metabolomics reveals altered waste clearance and accelerated aging in HIV patients with neurocognitive impairment. AIDS 28, 1579–1591. 10.1097/QAD.0000000000000303 24752083 PMC4086755

[B19] Chan S. J. Niu W. Hayakawa K. Hamanaka G. Wang X. Cheah P.. (2019). Promoting neuro-supportive properties of astrocytes with epidermal growth factor hydrogels. Stem Cells Transl. Med. 8, 1242–1248. 10.1002/sctm.19-0159 31483567 PMC6877762

[B20] Chauhan A. Mehla R. Vijayakumar T. S. Handy I. (2014). Endocytosis-mediated HIV-1 entry and its significance in the elusive behavior of the virus in astrocytes. Virology 45, 1–19. 10.1016/j.virol.2014.03.002 24889220 PMC4179455

[B21] Chen L. Khodr C. E. Al-Harthi L. Hu X. T. (2019). Aging and HIV-1 alter the function of specific K+ channels in prefrontal cortex pyramidal neurons. Neurosci. Lett. 708:134341. 10.1016/j.neulet.2019.134341 31255727 PMC6693957

[B22] Chen M. J. Sepramaniam S. Armugam A. Shyan Choy M. Manikandan J. Melendez A.. (2008). Water and ion channels: crucial in the initiation and progression of apoptosis in central nervous system? Curr. Neuropharmacol. 6, 102–116. 10.2174/157015908784533879 19305791 PMC2647147

[B23] Chmelova M. Sucha P. Bochin M. Vorisek I. Pivonkova H. Hermanova Z.. (2019). The role of aquaporin-4 and transient receptor potential vaniloid isoform 4 channels in the development of cytotoxic edema and associated extracellular diffusion parameter changes. Eur. J. Neurosci. 50, 1685–1699. 10.1111/ejn.14338 30633415

[B24] Churchill M. J. Cowley D. J. Wesselingh S. L. Gorry P. R. Gray L. R. (2015). HIV-1 transcriptional regulation in the central nervous system and implications for HIV cure research. J Neurovirol. 21, 290–300. 10.1007/s13365-014-0271-5 25060300 PMC4305497

[B25] Ciappelloni S. Bouchet D. Dubourdieu N. Boué-Grabot E. Kellermayer B. Manso C.. (2019). Aquaporin-4 surface trafficking regulates astrocytic process motility and synaptic activity in health and autoimmune disease. Cell Rep. 27, 3860.e4–3872.e4. 10.1016/j.celrep.2019.05.097 31242419

[B26] Ciccarelli N. (2019). Considerations on nosology for HIV-associated neurocognitive disorders: it is time to update? Infection 48, 1–6. 10.1007/s15010-019-01373-8 31691905

[B27] Clark D. P. Perreau V. M. Shultz S. R. Brady R. D. Lei E. Dixit S.. (2019). Inflammation in traumatic brain injury: roles for toxic A1 astrocytes and microglial-astrocytic crosstalk. Neurochem. Res. 44, 1410–1424. 10.1007/s11064-019-02721-8 30661228

[B28] Clarke L. E. Liddelow S. A. Chakraborty C. Münch A. E. Heiman M. Barres B. A. (2018). Normal aging induces A1-like astrocyte reactivity. Proc. Natl. Acad. Sci. U.S.A. 115, E1896–E1905. 10.1073/pnas.1800165115 29437957 PMC5828643

[B29] Cohen J. Torres C. (2019). Astrocyte senescence: evidence and significance. Aging Cell. 18:e12937. 10.1111/acel.12937 30815970 PMC6516680

[B30] Conant K. Tornatore C. Atwood W. Meyers K. Traub R. Major E.. (1994). *In vivo* and *in vitro* infection of the astrocyte by HIV-1. Adv. Neuroimmunol. 4, 287–289. 10.1016/S0960-5428(06)80269-X 7874397

[B31] Cysique L. A. Brew B. J. (2019). Comorbid depression and apathy in HIV-associated neurocognitive disorders in the era of chronic HIV infection. Handb. Clin. Neurol. 165, 71–82. 10.1016/B978-0-444-64012-3.00006-X 31727231

[B32] Da Mesquita S. Fu Z. Kipnis J. (2018a). The meningeal lymphatic system: a new player in neurophysiology. Neuron 100, 375–388. 10.1016/j.neuron.2018.09.022 30359603 PMC6268162

[B33] Da Mesquita S. Louveau A. Vaccari A. Smirnov I. Cornelison R. C. Kingsmore K. M.. (2018b). Functional aspects of meningeal lymphatics in ageing and Alzheimer's disease. Nature 560, 185–191. 10.1038/s41586-018-0368-8 30046111 PMC6085146

[B34] Dalkara T. Gursoy-Ozdemir Y. Yemisci M. (2011). Brain microvascular pericytes in health and disease. Acta Neuropathol. 122:1. 10.1007/s00401-011-0847-6 21656168

[B35] Damkier H. H. Brown P. D. Praetorius J. (2013). Cerebrospinal fluid secretion by the choroid plexus. Physiol. Rev. 93, 1847–1892. 10.1152/physrev.00004.2013 24137023

[B36] Dasdelen D. Mogulkoc R. Baltaci A. K. (2019). Aquaporins in brain health and brain injury. Mini. Rev. Med. Chem. 20, 498–512. 10.2174/1389557519666191018142007 31656150

[B37] Dave R. S. Jain P. Byrareddy S. N. (2018a). Follicular dendritic cells of lymph nodes as human immunodeficiency virus/simian immunodeficiency virus reservoirs and insights on cervical lymph node. Front. Immunol. 9:805. 10.3389/fimmu.2018.00805 29725333 PMC5916958

[B38] Dave R. S. Sharma R. K. Muir R. R. Haddad E. Gumber S. Villinger F.. (2018b). FDC:TFH interactions within cervical lymph nodes of SIV-infected rhesus macaques. J. Neuroimmune Pharmacol. 13, 204–218. 10.1007/s11481-017-9775-0 29288344 PMC5757373

[B39] De Leon M. J. Li Y. Okamura N. Tsui W. H. Saint-Louis L. A. Glodzik L.. (2017). Cerebrospinal fluid clearance in Alzheimer disease measured with dynamic PET. J. Nucl. Med. 58, 1471–1476. 10.2967/jnumed.116.187211 28302766 PMC5577629

[B40] Diop O. M. Gueye A. Dias-Tavares M. Kornfeld C. Faye A. Ave P.. (2000). High levels of viral replication during primary simian immunodeficiency virus SIVagm infection are rapidly and strongly controlled in African green monkeys. J. Virol. 74, 7538–7547. 10.1128/JVI.74.16.7538-7547.2000 10906207 PMC112274

[B41] Do T. Murphy G. Earl L. A. Del Prete G. Q. Grandinetti G. Li G. H.. (2014). Three-dimensional imaging of HIV-1 virological synapses reveals membrane architectures involved in virus transmission. J. Virol. 88, 10327–10339. 10.1128/JVI.00788-14 24965444 PMC4178837

[B42] Duvernoy H. M. Risold P. Y. (2007). The circumventricular organs: an atlas of comparative anatomy and vascularization. Brain Res. Rev. 56, 119–147. 10.1016/j.brainresrev.2007.06.002 17659349

[B43] Eide P. K. Vatnehol S. A. S. Emblem K. E. Ringstad G. (2018). Magnetic resonance imaging provides evidence of glymphatic drainage from human brain to cervical lymph nodes. Sci. Rep. 8, 7194–7194. 10.1038/s41598-018-25666-4 29740121 PMC5940793

[B44] Ferrell D. Giunta B. (2014). The impact of HIV-1 on neurogenesis: implications for HAND. Cell Mol. Life Sci. 71, 4387–4392. 10.1007/s00018-014-1702-4 25134912 PMC4224299

[B45] Ferrer I. (2017). Diversity of astroglial responses across human neurodegenerative disorders and brain aging. Brain Pathol. 27, 645–674. 10.1111/bpa.12538 28804999 PMC8029391

[B46] Filippidis A. S. Carozza R. B. Rekate H. L. (2016). Aquaporins in brain Edema and neuropathological conditions. Int. J. Mol. Sci. 18:55. 10.3390/ijms18010055 28036023 PMC5297690

[B47] Fletcher C. V. Staskus K. Wietgrefe S. W. Rothenberger M. Reilly C. Chipman J. G.. (2014). Persistent HIV-1 replication is associated with lower antiretroviral drug concentrations in lymphatic tissues. Proc. Natl. Acad. Sci. U.S.A. 111, 2307–2312. 10.1073/pnas.1318249111 24469825 PMC3926074

[B48] Frigeri A. Nicchia G. P. Verbavatz J. M. Valenti G. Svelto M. (1998). Expression of aquaporin-4 in fast-twitch fibers of mammalian skeletal muscle. J. Clin. Invest. 102, 695–703. 10.1172/JCI2545 9710437 PMC508931

[B49] Gaberel T. Gakuba C. Goulay R. de Lizarrondo S. M. Hanouz J. L. Emery E.. (2014). Impaired glymphatic perfusion after strokes revealed by contrast-enhanced MRI: a new target for fibrinolysis? Stroke 45, 3092–3096. 10.1161/STROKEAHA.114.006617 25190438

[B50] Gao Q. Li Y. Chopp M. (2005). Bone marrow stromal cells increase astrocyte survival via upregulation of phosphoinositide 3-kinase/threonine protein kinase and mitogen-activated protein kinase kinase/extracellular signal-regulated kinase pathways and stimulate astrocyte trophic factor gene expression after anaerobic insult. Neuroscience 136, 123–134. 10.1016/j.neuroscience.2005.06.091 16198497

[B51] Golanov E. V. Bovshik E. I. Wong K. K. Pautler R. G. Foster C. H. Federley R. G.. (2018). Subarachnoid hemorrhage - induced block of cerebrospinal fluid flow: role of brain coagulation factor III (tissue factor). J. Cereb. Blood Flow Metab. 38, 793–808. 10.1177/0271678X17701157 28350198 PMC5987942

[B52] Goldstein S. Ourmanov I. Brown C. R. Beer B. E. Elkins W. R. Plishka R.. (2000). Wide range of viral load in healthy african green monkeys naturally infected with simian immunodeficiency virus. J. Virol. 74, 11744–11753. 10.1128/JVI.74.24.11744-11753.2000 11090174 PMC112457

[B53] Goulay R. Flament J. Gauberti M. Naveau M. Pasquet N. Gakuba C.. (2017). Subarachnoid hemorrhage severely impairs brain parenchymal cerebrospinal fluid circulation in nonhuman primate. Stroke 48, 2301–2305. 10.1161/STROKEAHA.117.017014 28526764

[B54] Guaraldi G. Milic J. Mussini C. (2019). Aging with HIV. Curr HIV/AIDS Rep. 16, 475–481. 10.1007/s11904-019-00464-3 31823125

[B55] Guo M. L. Buch S. (2019). Neuroinflammation & pre-mature aging in the context of chronic HIV infection and drug abuse: role of dysregulated autophagy. Brain Res. 1724:146446. 10.1016/j.brainres.2019.146446 31521638 PMC6933726

[B56] Haddow L. J. Floyd S. A. Copas Gilson R. J. (2013). A systematic review of the screening accuracy of the HIV dementia scale and International HIV dementia scale. PLoS ONE 8:e61826. 10.1371/journal.pone.0061826 23613945 PMC3628906

[B57] Harder D. R. Zhang C. Gebremedhin D. (2002). Astrocytes function in matching blood flow to metabolic activity. News Physiol. Sci. 17, 27–31. 10.1152/physiologyonline.2002.17.1.27 11821533

[B58] Hategan A. Masliah E. Nath A. (2019). HIV and Alzheimer's disease: complex interactions of HIV-Tat with amyloid beta peptide and Tau protein. J. Neurovirol. 25, 648–660. 10.1007/s13365-019-00736-z 31016584 PMC9056081

[B59] Hayakawa K. Pham L. D. Arai K. Lo E. H. (2014). Reactive astrocytes promote adhesive interactions between brain endothelium and endothelial progenitor cells via HMGB1 and beta-2 integrin signaling. Stem Cell Res. 12, 531–538. 10.1016/j.scr.2013.12.008 24480450 PMC3966549

[B60] Heesters B. A. Lindqvist M. Vagefi P. A. Scully E. P. (2015). Follicular dendritic cells retain infectious HIV in cycling endosomes. PLoS Pathog. 11:e1005285. 10.1371/journal.ppat.1005285 26623655 PMC4666623

[B61] Hinkle J. T. Dawson V. L. Dawson T. M. (2019). The A1 astrocyte paradigm: new avenues for pharmacological intervention in neurodegeneration. Mov. Disord. 34, 959–969. 10.1002/mds.27718 31136698 PMC6642014

[B62] Hinson S. R. Roemer S. F. Lucchinetti C. F. Fryer J. P. Kryzer T. J. Chamberlain J. L.. (2008). Aquaporin-4-binding autoantibodies in patients with neuromyelitis optica impair glutamate transport by down-regulating EAAT2. J. Exp. Med. 205, 2473–2481. 10.1084/jem.20081241 18838545 PMC2571922

[B63] Ho J. Moir S. Kulik L. Malaspina A. Donoghue E. T. Miller N. J.. (2007). Role for CD21 in the establishment of an extracellular HIV reservoir in lymphoid tissues. J. Immunol. 178, 6968–6974. 10.4049/jimmunol.178.11.6968 17513746

[B64] Hong S. Banks W. A. (2015). Role of the immune system in HIV-associated neuroinflammation and neurocognitive implications. Brain Behav. Immun. 45, 1–12. 10.1016/j.bbi.2014.10.008 25449672 PMC4342286

[B65] Hubbard J. A. Szu J. I. Binder D. K. (2018). The role of aquaporin-4 in synaptic plasticity, memory and disease. Brain Res. Bull. 136, 118–129. 10.1016/j.brainresbull.2017.02.011 28274814

[B66] Huda S. Whittam D. Bhojak M. Chamberlain J. Noonan C. Jacob A. (2019). Neuromyelitis optica spectrum disorders. Clin. Med. 19, 169–176. 10.7861/clinmedicine.19-2-169 30872305 PMC6454358

[B67] Huot N. Bosinger S. E. Paiardini M. Reeves R. K. Muller-Trutwin M. (2018). Lymph node cellular and viral dynamics in natural hosts and impact for HIV cure strategies. Front. Immunol. 9:780. 10.3389/fimmu.2018.00780 29725327 PMC5916971

[B68] Huot N. Rascle P. Garcia-Tellez T. Jacquelin B. Muller-Trutwin M. (2016). Innate immune cell responses in non pathogenic versus pathogenic SIV infections. Curr. Opin. Virol. 19, 37–44. 10.1016/j.coviro.2016.06.011 27447445

[B69] Iliff J. J. Chen M. J. Plog B. A. Zeppenfeld D. M. Soltero M. Yang L.. (2014). Impairment of glymphatic pathway function promotes tau pathology after traumatic brain injury. J. Neurosci. 34, 16180–16193. 10.1523/JNEUROSCI.3020-14.2014 25471560 PMC4252540

[B70] Iliff J. J. Goldman S. A. Nedergaard M. (2015). Implications of the discovery of brain lymphatic pathways. Lancet Neurol. 14, 977–979. 10.1016/S1474-4422(15)00221-5 26376966 PMC4655610

[B71] Iliff J. J. Lee H. Yu M. Feng T. Logan J. Nedergaard M.. (2013). Brain-wide pathway for waste clearance captured by contrast-enhanced MRI. J. Clin. Invest. 123, 1299–1309. 10.1172/JCI67677 23434588 PMC3582150

[B72] Iliff J. J. Wang M. Liao Y. Plogg B. A. Peng W. Gundersen G. A.. (2012). A paravascular pathway facilitates CSF flow through the brain parenchyma and the clearance of interstitial solutes, including amyloid β. Sci. Transl. Med. 4:147ra111. 10.1126/scitranslmed.3003748 22896675 PMC3551275

[B73] Jessen N. A. Munk A. Lundgaard S. I. Nedergaard M. (2015). The glymphatic system: a beginner's guide. Neurochem. Res. 40, 2583–2599. 10.1007/s11064-015-1581-6 25947369 PMC4636982

[B74] Joshi A. U. Minhas P. S. Liddelow S. A. Haileselassie B. Andreasson K. I. Dorn G. W. 2nd. (2019). Fragmented mitochondria released from microglia trigger A1 astrocytic response and propagate inflammatory neurodegeneration. Nat. Neurosci. 22, 1635–1648. 10.1038/s41593-019-0486-0 31551592 PMC6764589

[B75] Jung E. Gardner D. Choi D. Park E. Jin Seong Y. Yang S.. (2017). Development and characterization of a novel Prox1-EGFP lymphatic and schlemm's canal reporter rat. Sci. Rep. 7:5577. 10.1038/s41598-017-06031-3 28717161 PMC5514086

[B76] Koehler R. C. Gebremedhin D. Harder D. R. (2006). Role of astrocytes in cerebrovascular regulation. J. Appl. Physiol. 100, 307–317. 10.1152/japplphysiol.00938.2005 16357084 PMC1819408

[B77] Kress B. T. Iliff J. J. Xia M. Wang M. Wei H. S. Zeppenfeld D.. (2014). Impairment of paravascular clearance pathways in the aging brain. Ann. Neurol. 76, 845–861. 10.1002/ana.24271 25204284 PMC4245362

[B78] Li C. Zhao R. Gao K. Wei Z. Yaoyao Yin M. Ting Lau L.. (2011). Astrocytes: implications for neuroinflammatory pathogenesis of Alzheimer's disease. Curr. Alzheimer Res. 8, 67–80. 10.2174/156720511794604543 21143158

[B79] Liao S. Padera T. P. (2013). Lymphatic function and immune regulation in health and disease. Lymphat. Res. Biol. 11, 136–143. 10.1089/lrb.2013.0012 24024577 PMC3780287

[B80] Liddelow S. A. Barres B. A. (2017). Reactive astrocytes: production, function, and therapeutic potential. Immunity 46, 957–967. 10.1016/j.immuni.2017.06.006 28636962

[B81] Liddelow S. A. Guttenplan K. A. Clarke L. E. Bennett F. C. Bohlen C. J. Schirmer L.. (2017). Neurotoxic reactive astrocytes are induced by activated microglia. Nature 541, 481–487. 10.1038/nature21029 28099414 PMC5404890

[B82] Louveau A. Smirnov I. Keyes T. J. Eccles J. D. Rouhani S. J. Peske J. D.. (2015). Structural and functional features of central nervous system lymphatic vessels. Nature 523, 337–341. 10.1038/nature14432 26030524 PMC4506234

[B83] Lundgaard I. Lu M. L. Yang E. Peng W. Mestre H. Hitomi E.. (2017). Glymphatic clearance controls state-dependent changes in brain lactate concentration. J. Cereb. Blood Flow Metab. 37, 2112–2124. 10.1177/0271678X16661202 27481936 PMC5464705

[B84] Luo X. He J. J. (2015). Cell-cell contact viral transfer contributes to HIV infection and persistence in astrocytes. J. Neurovirol. 21, 66–80. 10.1007/s13365-014-0304-0 25522787 PMC4861053

[B85] Lutgen V. Narasipura S. D. Barbian H. J. Richards M. Wallace J. Razmpour R.. (2020). HIV infects astrocytes *in vivo* and egresses from the brain to the periphery. PLoS Pathog. 16:e1008381. 10.1371/journal.ppat.1008381 32525948 PMC7289344

[B86] Lv Y. Huang Q. Dai W. Jie Y. Yu G. Fan X.. (2018). AQP9 promotes astrocytoma cell invasion and motility via the AKT pathway. Oncol. Lett. 16, 6059–6064. 10.3892/ol.2018.9361 30344749 PMC6176364

[B87] Ma T. H. Gao H. W. Fang X. D. Yang H. (2011). Expression and function of aquaporins in peripheral nervous system. Acta Pharmacol. Sin. 32, 711–715. 10.1038/aps.2011.63 21602841 PMC4009970

[B88] Mackiewicz M. M. Overk C. Achim C. L. Masliah E. (2019). Pathogenesis of age-related HIV neurodegeneration. J. Neurovirol. 25, 622–633. 10.1007/s13365-019-00728-z 30790184 PMC6703984

[B89] Mader S. Brimberg L. (2019). Aquaporin-4 water channel in the brain and its implication for health and disease. Cells 8:90. 10.3390/cells8020090 30691235 PMC6406241

[B90] Masjedi A. Hassannia H. Atyabi F. Rastegari A. Hojjat-Farsangi M. Namdar A.. (2019). Downregulation of A2AR by siRNA loaded PEG-chitosan-lactate nanoparticles restores the T cell mediated anti-tumor responses through blockage of PKA/CREB signaling pathway. Int J. Biol. Macromol. 133, 436–445. 10.1016/j.ijbiomac.2019.03.223 30936011

[B91] Matchanova A. Woods S. P. Kordovski V. M. (2019). Operationalizing and evaluating the Frascati criteria for functional decline in diagnosing HIV-associated neurocognitive disorders in adults. J. Neurovirol. 26, 1–13. 10.1007/s13365-019-00809-z 31745823 PMC7234924

[B92] Matias I. Morgado J. Gomes F. C. A. (2019). Astrocyte heterogeneity: impact to brain aging and disease. Front. Aging Neurosci. 11:59. 10.3389/fnagi.2019.00059 30941031 PMC6433753

[B93] Mealy M. A. Wingerchuk D. M. Palace J. Greenberg B. M. Levy M. (2014). Comparison of relapse and treatment failure rates among patients with neuromyelitis optica: multicenter study of treatment efficacy. JAMA Neurol. 71, 324–330. 10.1001/jamaneurol.2013.5699 24445513

[B94] Mestre H. Hablitz L. M. Xavier A. L. Feng W.. (2018). Aquaporin-4-dependent glymphatic solute transport in the rodent brain. Elife 7:e40070. 10.7554/eLife.40070.022 30561329 PMC6307855

[B95] Métral M. Darling K. Locatelli I. Nadin I. Santos G. Brugger P.. (2020). The neurocognitive assessment in the metabolic and aging cohort (NAMACO) study: baseline participant profile. HIV Med. 21, 30–42. 10.1111/hiv.12795 31589807 PMC6916574

[B96] Misawa T. Arima K. Mizusawa H. Satoh J. (2008). Close association of water channel AQP1 with amyloid-beta deposition in Alzheimer disease brains. Acta Neuropathol. 116, 247–260. 10.1007/s00401-008-0387-x 18509662 PMC2516196

[B97] Nakada T. Kwee I. L. Igarashi H. Suzuki Y. (2017). Aquaporin-4 functionality and virchow-robin space water dynamics: physiological model for neurovascular coupling and glymphatic flow. Int. J. Mol. Sci. 18:1798. 10.3390/ijms18081798 28820467 PMC5578185

[B98] Narasipura S. D. Henderson L. J. Fu S. W. Chen L. Kashanchi F. Al-Harthi L. (2012). Role of beta-catenin and TCF/LEF family members in transcriptional activity of HIV in astrocytes. J. Virol. 86, 1911–1921. 10.1128/JVI.06266-11 22156527 PMC3302377

[B99] Olivier I. S. Cacabelos R. Naidoo V. (2018). Risk factors and pathogenesis of HIV-associated neurocognitive disorder: the role of host genetics. Int. J. Mol. Sci. 19:3594. 10.3390/ijms19113594 30441796 PMC6274730

[B100] Onanga R. Kornfeld C. Pandrea I. Estaquier J. Souquiere S. Rouquet P.. (2002). High levels of viral replication contrast with only transient changes in CD4(+) and CD8(+) cell numbers during the early phase of experimental infection with simian immunodeficiency virus SIVmnd-1 in Mandrillus sphinx. J. Virol. 76, 10256–10263. 10.1128/JVI.76.20.10256-10263.2002 12239301 PMC136548

[B101] Orr A. G. Hsiao E. C. Wang M. M. Ho K. Kim D. H. Wang X.. (2015). Astrocytic adenosine receptor A2A and Gs-coupled signaling regulate memory. Nat. Neurosci. 18, 423–434. 10.1038/nn.3930 25622143 PMC4340760

[B102] Paiardini M. Muller-Trutwin M. (2013). HIV-associated chronic immune activation. Immunol. Rev. 254, 78–101. 10.1111/imr.12079 23772616 PMC3729961

[B103] Papadopoulos M. C. Verkman A. S. (2013). Aquaporin water channels in the nervous system. Nat. Rev. Neurosci. 14, 265–277. 10.1038/nrn3468 23481483 PMC3732112

[B104] Pérez E. Barrachina M. Rodríguez A. Torrejón-Escribano B. Boada M. Hernández I.. (2007). Aquaporin expression in the cerebral cortex is increased at early stages of Alzheimer disease. Brain Res. 1128, 164–174. 10.1016/j.brainres.2006.09.109 17123487

[B105] Plog B. A. Dashnaw M. L. Hitomi E. Peng W. Liao Y. Lou N.. (2015). Biomarkers of traumatic injury are transported from brain to blood via the glymphatic system. J. Neurosci. 35, 518–526. 10.1523/JNEUROSCI.3742-14.2015 25589747 PMC4293408

[B106] Plog B. A. Nedergaard M. (2018). The glymphatic system in central nervous system health and disease: past, present, and future. Annu. Rev. Pathol. 13, 379–394. 10.1146/annurev-pathol-051217-111018 29195051 PMC5803388

[B107] Pluta R. Bogucka-Kocka A. Ułamek-Kozioł M. Bogucki J. Januszewski S. Kocki J. (2018a). Ischemic tau protein gene induction as an additional key factor driving development of Alzheimer's phenotype changes in CA1 area of hippocampus in an ischemic model of Alzheimer's disease. Pharmacol. Rep. 70, 881–884. 10.1016/j.pharep.2018.03.004 30096486

[B108] Pluta R. Ulamek-Koziol M. Januszewski S. Czuczwar S. J. (2018b). Tau protein dysfunction after brain Ischemia. J. Alzheimers Dis. 66, 429–437. 10.3233/JAD-180772 30282370 PMC6218135

[B109] Potokar M. J. Jorgačevski Zorec R. (2016). Astrocyte aquaporin dynamics in health and disease. Int. J. Mol. Sci. 17:1121. 10.3390/ijms17071121 27420057 PMC4964496

[B110] Rainey-Smith S. R. Mazzucchelli G. N. Villemagne V. L. Brown B. M. Porter T. Weinborn M.. (2018). Genetic variation in Aquaporin-4 moderates the relationship between sleep and brain Aβ-amyloid burden. Transl. Psychiatr. 8:47. 10.1038/s41398-018-0094-x 29479071 PMC5865132

[B111] Rasmussen M. K. Mestre H. Nedergaard M. (2018). The glymphatic pathway in neurological disorders. Lancet Neurol. 17, 1016–1024. 10.1016/S1474-4422(18)30318-1 30353860 PMC6261373

[B112] Redzic Z. (2011). Molecular biology of the blood-brain and the blood-cerebrospinal fluid barriers: similarities and differences. Fluids Barriers CNS 8:3. 10.1186/2045-8118-8-3 21349151 PMC3045361

[B113] Rey-Cuillé M. A. Berthier J. L. Bomsel-Demontoy M. C. Chaduc Y. Montagnier L. Hovanessian A. G.. (1998). Simian immunodeficiency virus replicates to high levels in sooty mangabeys without inducing disease. J. Virol. 72, 3872–3886. 10.1128/JVI.72.5.3872-3886.1998 9557672 PMC109612

[B114] Ringstad G. Valnes L. M. Dale A. M. Pripp A. H. Vatnehol S. A. S. Emblem K. E.. (2018). Brain-wide glymphatic enhancement and clearance in humans assessed with MRI. JCI Insight 3:e121537. 10.1172/jci.insight.121537 29997300 PMC6124518

[B115] Ringstad G. Vatnehol S. A. S. Eide P. K. (2017). Glymphatic MRI in idiopathic normal pressure hydrocephalus. Brain 140, 2691–2705. 10.1093/brain/awx191 28969373 PMC5841149

[B116] Robinson-Papp J. Gensler G. Navis A. Sherman S. Ellis R. J. Gelman B. B.. (2019). Characteristics of motor dysfunction in longstanding HIV. Clin. Infect. Dis. [Epub ahead of print]. 10.1093/cid/ciz986 31587032 PMC7486845

[B117] Rojas-Celis V. Valiente-Echeverría F. Soto-Rifo R. Toro-Ascuy D. (2019). New challenges of HIV-1 Infection: how HIV-1 attacks and resides in the central nervous system. Cells 8:1245. 10.3390/cells8101245 31614895 PMC6829584

[B118] Rosu G. C. Pirici I. Grigorie A. A. Istrate-Ofiteru A. M. Iovan L. Tudorica V.. (2019). Distribution of Aquaporins 1 and 4 in the central nervous system. Curr. Health Sci. J. 45, 218–226. 10.12865/CHSJ.45.02.14 31624651 PMC6778305

[B119] Russell R. A. Chojnacki J. Jones D. M. Johnson E. Do T. Eggeling C.. (2017). Astrocytes resist HIV-1 fusion but engulf infected macrophage material. Cell Rep. 18, 1473–1483. 10.1016/j.celrep.2017.01.027 28178524 PMC5316642

[B120] Sabri F. Prados A. Muñoz-Fernández R. Lantto R. Fernandez-Rubio P. Nasi A.. (2016). Impaired B cells survival upon production of inflammatory cytokines by HIV-1 exposed follicular dendritic cells. Retrovirology 13:61. 10.1186/s12977-016-0295-4 27596745 PMC5011926

[B121] Sakalauskaite-Juodeikiene E. Armaliene G. Kizlaitiene R. Bagdonaite L. Giedraitiene N. Mickevičiene D.. (2018). Detection of aquaporin-4 antibodies for patients with CNS inflammatory demyelinating diseases other than typical MS in Lithuania. Brain Behav 8:e01129. 10.1002/brb3.1129 30284401 PMC6236230

[B122] Saloner R. Campbell L. M. Serrano V. Montoya J. L. Pasipanodya E. Paolillo E. W.. (2019). Neurocognitive superaging in older adults living with HIV: demographic, neuromedical and everyday functioning correlates. J. Int. Neuropsychol. Soc. 25, 507–519. 10.1017/S1355617719000018 30890191 PMC6705613

[B123] Schain A. J. Melo-Carrillo A. Strassman A. M. Burstein R. (2017). Cortical spreading depression closes paravascular space and impairs glymphatic flow: implications for migraine headache. J. Neurosci. 37, 2904–2915. 10.1523/JNEUROSCI.3390-16.2017 28193695 PMC5354333

[B124] Shi Q. Wu Y. Z. Yang X. Xiao K. (2019). Significant enhanced expressions of aquaporin-1,−4 and−9 in the brains of various prion diseases. Prion 13, 173–184. 10.1080/19336896.2019.1660487 31814527 PMC6746548

[B125] Shijo M. Hamasaki H. Honda H. Suzuki S. O. Tachibana M. Ago T.. (2019). Upregulation of annexin A1 in reactive astrocytes and its subtle induction in microglia at the boundaries of human brain infarcts. J. Neuropathol. Exp. Neurol. 78, 961–970. 10.1093/jnen/nlz079 31504683

[B126] St Hillaire C. Vargas D. Pardo C. A. Gincel D. Mann J. Rothstein J. D.. (2005). Aquaporin 4 is increased in association with human immunodeficiency virus dementia: implications for disease pathogenesis. J. Neurovirol. 11, 535–543. 10.1080/13550280500385203 16338747

[B127] Stower H. (2018). Meningeal lymphatics in aging and Alzheimer's disease. Nat. Med. 24:1781. 10.1038/s41591-018-0281-6 30523314

[B128] Sun L. Zhang Y. Liu E. Ma Q. Anatol M. Han H. Yan J. (2019). The roles of astrocyte in the brain pathologies following ischemic stroke. Brain Inj 33, 712–716. 10.1080/02699052.2018.1531311 30335519

[B129] Sweeney A. M. Pla V. Du T. Liu G. Sun Q. Peng S.. (2019). *In vivo* imaging of cerebrospinal fluid transport through the intact mouse skull using fluorescence macroscopy. J. Vis. Exp. 10.3791/59774 31403617 PMC7001880

[B130] Thrane A. S. Rangroo Thrane V. Nedergaard M. (2014). Drowning stars: reassessing the role of astrocytes in brain edema. Trends Neurosci. 37, 620–628. 10.1016/j.tins.2014.08.010 25236348 PMC4253572

[B131] Valcour V. Sithinamsuwan P. Letendre S. Ances B. (2011). Pathogenesis of HIV in the central nervous system. Curr HIV/AIDS Rep. 8, 54–61. 10.1007/s11904-010-0070-4 21191673 PMC3035797

[B132] Winston A. Spudich S. (2020). Cognitive disorders in people living with HIV. Lancet HIV 7, e504–e513. 10.1016/S2352-3018(20)30107-7 32621876

[B133] Wu J. Ding D. Wang X. Li Q. Sun Y. Li L. Wang Y.. (2019). Regulation of aquaporin 4 expression by lipoxin A4 in astrocytes stimulated by lipopolysaccharide. Cell Immunol. 344:103959. 10.1016/j.cellimm.2019.103959 31383359

[B134] Xie L. Kang H. Xu Q. Chen M. J. Liao Y. Thiyagarajan M.. (2013). Sleep drives metabolite clearance from the adult brain. Science 342, 373–377. 10.1126/science.1241224 24136970 PMC3880190

[B135] Xing H. Q. Zhang Y. Izumo K. Arishima S. Kubota R. Ye X.. (2017). Decrease of aquaporin-4 and excitatory amino acid transporter-2 indicate astrocyte dysfunction for pathogenesis of cortical degeneration in HIV-associated neurocognitive disorders. Neuropathology 37, 25–34. 10.1111/neup.12321 27506782

[B136] Xu M. Xiao M. Li S. Yang B. (2017). Aquaporins in nervous system. Adv. Exp. Med. Biol. 969, 81–103. 10.1007/978-94-024-1057-0_5 28258567

[B137] Yang H. Y. Beymer M. R. Suen S. C. (2019). Chronic disease onset among people living with HIV and AIDS in a large private insurance claims dataset. Sci. Rep. 9:18514. 10.1038/s41598-019-54969-3 31811207 PMC6897968

[B138] Yool A. J. (2007). Aquaporins: multiple roles in the central nervous system. Neuroscientist 13, 470–485. 10.1177/1073858407303081 17901256

[B139] Zador Z. Stiver S. Wang V. Manley G. T. (2009). Role of aquaporin-4 in cerebral edema and stroke. Handb. Exp. Pharmacol. 2009:159–170. 10.1007/978-3-540-79885-9_7 19096776 PMC3516842

[B140] Zamanian J. L. Xu L. Foo L. C. Nouri N. Zhou L. Giffard R. G.. (2012). Genomic analysis of reactive astrogliosis. J. Neurosci. 32, 6391–6410. 10.1523/JNEUROSCI.6221-11.2012 22553043 PMC3480225

[B141] Zbesko J. C. Nguyen T. V. V. Yang T. Frye J. B. Hussain O. Hayes M.. (2018). Glial scars are permeable to the neurotoxic environment of chronic stroke infarcts. Neurobiol. Dis. 112, 63–78. 10.1016/j.nbd.2018.01.007 29331263 PMC5851450

[B142] Zhang Y. Xu K. Liu Y. Erokwu B. O. Zhao P. Flask C. A.. (2019). Increased cerebral vascularization and decreased water exchange across the blood-brain barrier in aquaporin-4 knockout mice. PLoS ONE 14:e0218415. 10.1371/journal.pone.0218415 31220136 PMC6586297

[B143] Zhao Z. A. Li P. Ye S. Y. Ning Y. L. Wang H. Peng Y.. (2017). Perivascular AQP4 dysregulation in the hippocampal CA1 area after traumatic brain injury is alleviated by adenosine A2A receptor inactivation. Sci. Rep. 7:2254. 10.1038/s41598-017-02505-6 28533515 PMC5440401

